# Prognostic Impact of Pretherapeutic FDG-PET in Localized Anal Cancer

**DOI:** 10.3390/cancers12061512

**Published:** 2020-06-09

**Authors:** Maelle Le Thiec, Aude Testard, Ludovic Ferrer, Camille Guillerminet, Olivier Morel, Bruno Maucherat, Daniela Rusu, Sylvie Girault, Marie Lacombe, Hadji Hamidou, Véronique Guérin Meyer, Emmanuel Rio, Sandrine Hiret, Françoise Kraeber-Bodéré, Loïc Campion, Caroline Rousseau

**Affiliations:** 1 Nuclear Medicine Unit, ICO Cancer Center, 44805 Saint Herblain, France; Bruno.Maucherat@ico.unicancer.fr (B.M.); Daniela.Rusu@ico.unicancer.fr (D.R.); Francoise.Bodere@ico.unicancer.fr (F.K.-B.); Caroline.Rousseau@ico.unicancer.fr (C.R.); 2 Nuclear Medicine Unit, ICO Cancer Center, 49055 Angers, France; Aude.Testard@ico.unicancer.fr (A.T.); Olivier.Morel@ico.unicancer.fr (O.M.); Sylvie.Girault@ico.unicancer.fr (S.G.); Marie.Lacombe@ico.unicancer.fr (M.L.); 3 Medical Physics Unit, ICO Cancer Center, 44805 Saint Herblain, France; Ludovic.Ferrer@ico.unicancer.fr; 4 CRCINA, University of Nantes and Angers, INSERM UMR1232, CNRS-ERL6001, 49055 Angers, France; Loic.Campion@ico.unicancer.fr; 5 Medical Physics Unit, ICO Cancer Center, 49055 Angers, France; Camille.Guillerminet@ico.unicancer.fr; 6 Radiation Oncology Unit, ICO Cancer Center, 49055 Angers, France; Hadji.Hamidou@ico.unicancer.fr; 7 Medical oncology Unit, ICO Cancer Center, 49055 Angers, France; Veronique.Guerin-Meyer@ico.unicancer.fr; 8 Radiation Oncology Unit, ICO Cancer Center, 44805 Saint Herblain, France; Emmanuel.Rio@ico.unicancer.fr; 9 Medical oncology Unit, ICO Cancer Center, 44805 Saint Herblain, France; Sandrine.Hiret@ico.unicancer.fr; 10 Biometrics Unit, ICO Cancer Center, 44805 Saint Herblain, France

**Keywords:** anal cancer, FDG-PET, prognosis, metabolic tumour volume

## Abstract

Due to the heterogeneity of tumour mass segmentation methods and lack of consensus, our study evaluated the prognostic value of pretherapeutic positron emission tomography with fluorodeoxyglucose (FDG-PET) metabolic parameters using different segmentation methods in patients with localized anal squamous cell carcinoma (SCC). Eighty-one patients with FDG-PET before radiochemotherapy were retrospectively analyzed. Semiquantitative data were measured with three fixed thresholds (35%, 41% and 50% of Maximum Standardized Uptake Value (SUVmax)) and four segmentation methods based on iterative approaches (Black, Adaptive, Nestle and Fitting). Metabolic volumes of primary anal tumour (P-MTV) and total tumour load (T-MTV: P-MTV+ lymph node MTV) were calculated. The primary endpoint was event-free survival (EFS). Seven multivariate models were created to compare FDG-PET tumour volumes prognostic impact. For all segmentation thresholds, PET metabolic volume parameters were independent prognostic factor and T-MTV variable was consistently better associated with EFS than P-MTV. Patient’s sex was an independent variable and significantly correlated with EFS. With fixed threshold segmentation methods, 35% of SUVmax threshold seemed better correlated with EFS and the best cut-off for discrimination between a low and high risk of event occurrence was 40 cm^3^. Determination of T-MTV by FDG-PET using fixed threshold segmentation is useful for predicting EFS for primary anal SCC. If these data are confirmed in larger studies, FDG-PET could contribute to individualized patient therapies.

## 1. Introduction

Anal squamous cell carcinoma (SCC) is a rare tumour that accounts for only 1.5% of digestive cancers, but its incidence has increased in recent decades [1,2,3,4,5]. More than 90% of patients present with locoregional disease at diagnosis and the standard treatment are based on radiochemotherapy [6,7,8,9]. This therapeutic approach for locally advanced anal SCC has led to a five-year overall survival (OS) ranging from 61% to 85% [10]. It is therefore important to identify patients who are more likely to have disease recurrence and progression, so that immediate and specific therapies can be implemented at diagnosis in order to obtain a better disease prognosis. The most significant known clinical prognostic factors are the size of the primary tumour and the initial loco-regional node invasion [11,12,13,14,15,16,17,18]. Positron emission tomography with fluorodeoxyglucose (FDG-PET) is recommended for the initial staging of anal SCC with detection of inguinal and pelvic lymph node involvement [19,20,21,22,23,24,25,26,27]. In recent years, several teams have investigated the prognostic value of semiquantitative PET data during pretherapy, post-therapy and the metabolic response phases of this pathology [20,28,29,30,31,32,33,34]. While these studies showed a prognostic value for the MTV (metabolic tumour volume) using pretherapeutic FDG-PET, the methods for tumour mass segmentation are heterogeneous, with different fixed thresholds because of easier routine clinical use and the absence of a standardized method. Other segmentation methods have been proposed, such as those based on iterative approaches to determine the optimal threshold [35,36,37,38,39], but there is no data to support this in this indication. The aim of our study was to evaluate the prognostic value of pretherapeutic FDG-PET metabolic parameters for patients with localized or locally advanced anal SCC in order to improve personalized care for patients with a risk of recurrence.

## 2. Results

### 2.1. Patient Population

The characteristics of the 81 patients included in the study are described in Table 1. The node staging was modified in 34.6% of patients with a node upstaging in 29.6%: 63 patients with a node stage N0 or N1 by conventional imaging were up-staged in N2 for 11 of them (17.5%) and N3 for 7 (11%). The median duration of follow-up was 3.3 years (2.5–5 years). At the end of the follow-up, 55 patients (67.9%) were still alive without signs of recurrence. Twenty-six patients (32%) had an event, 9 of which had metastatic progression and 15 died (11 specific deaths, including 5 patients at metastatic stage, and 4 died from another cause but after recurrence of their disease). The 1-year event-free survival (EFS) was 76.5% and was 70.4% at 2 years.

### 2.2. Univariate Analysis

The results are presented in Table 2. Among the clinical, conventional imaging, and FDG-PET data, male status was associated with a more pejorative EFS (*p* < 0.007). HIV status was not analyzed because only two patients were HIV positive.

Detection of a pelvic lymph node lesion was not associated with a pejorative EFS if described on FDG-PET imaging (*p* = 0.092—trend), nor if detection is done by conventional imaging (*p* = 0.235). Bilateral pelvic lymph node involvement diagnosed by FDG-PET was associated with pejorative EFS (*p* = 0.018), and there was a similar trend if inguinal lymph node involvement was detected by FDG-PET (*p* = 0.056).

In the quantitative FDG-PET data analysis, no significant association was found between the SUVmax (maximum Standardized Uptake Value, which corresponds to the voxel with the maximum activity concentration within the tumour scaled by the administered activity) of the primary tumour and the EFS (*p* = 0.257). Independent of the segmentation thresholding method, the metabolic volume parameters (P-MTV and T-MTV calculated as the sum of P-MTV and lymph node MTV) were significantly associated with a negative EFS (Table 2).

### 2.3. Multivariate Analysis

Similar to the univariate analysis, the PET metabolic volume parameters were significantly associated with EFS and were independent of the segmentation threshold method. Seven multivariate models (one per threshold type) were created to compare the prognostic impact of the different tumour volumes defined on FDG-PET. Each model included the two clinically significant univariate variables (the patient’s sex and the presence of bilateral pelvic lymph node involvement on FDG-PET) and one of the tumour metabolic volumes defined by a segmentation method (Table 3).

Regardless of the model, the patient’s sex remained an independent prognostic factor for EFS, which was not the case for the presence of bilateral pelvic lymph node involvement on FDG-PET. For each threshold, each metabolic tumour volume (P-MTV and T-MTV) remained an independent prognostic factor.

After AIC comparison of P-MTV and T-MTV prognostic performance in each threshold, the T-MTV variable was consistently better than the P-MTV variable. By only considering the segmentation methods according to a fixed threshold (35%, 41%, 50% of SUVmax), the 35% of SUVmax threshold correlated the strongest with EFS. If we considered the segmentation methods based on iterative approaches (Black, Adaptative, Nestlé and Fitting), the set of methods gave consistent and homogenous prognostic results comparable to the 35% SUVmax threshold results.

**Figure 1:**
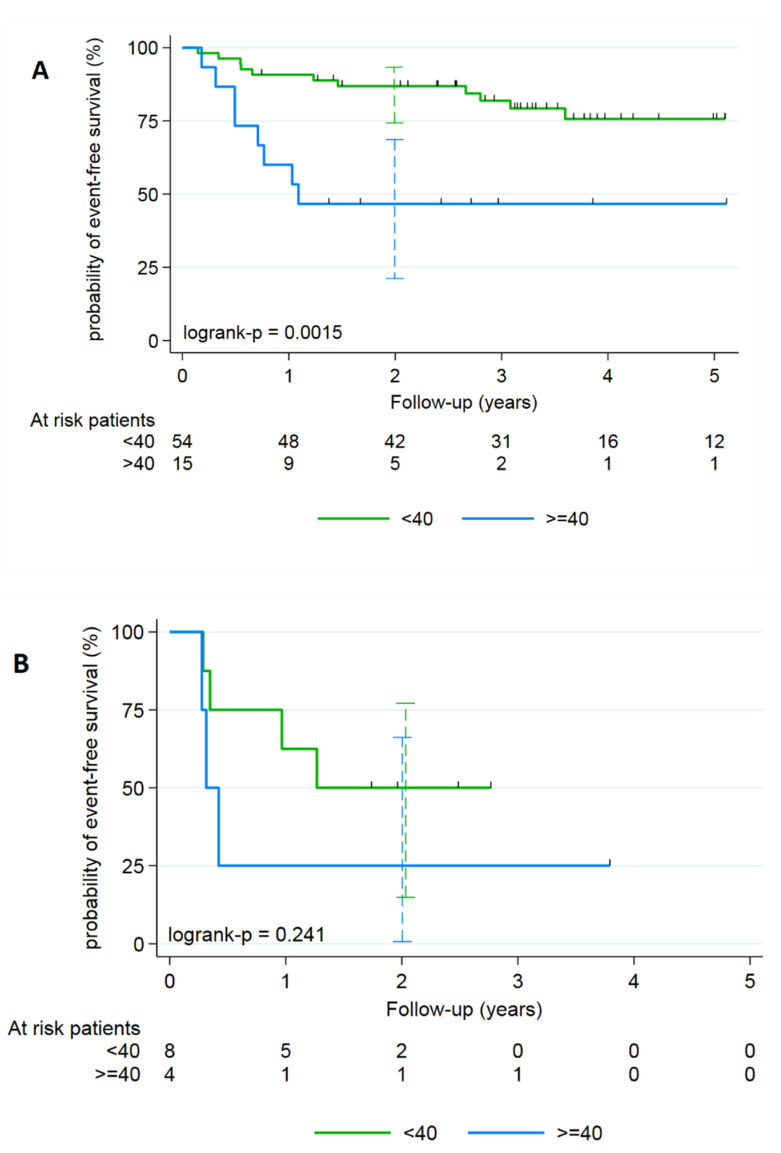
Kaplan–Meier analyzes of the EFS for anal SCC according to the T-MTV defined with a segmentation of 35% of the SUVmax threshold and stratified on gender: (**A**) female population and (**B**) male.

As it was easier to determine T-MTV by FDG-PET using a fixed 35% threshold segmentation than using an iterative segmentation method without losing significant efficiency in EFS prediction, this model was kept. To make it easier to interpret this continuous parameter in clinical practice, a binary categorization based on an optimal cutoff value was done using the Contal and O’Quigley method. The best discrimination cut-off between low- and high-risk event occurrence was 40 cm^3^ with a 35% of SUVmax threshold. As it was difficult to split our population into a training and a testing sets because of the small number of events (*n* = 26), robustness of the parameter was tested and confirmed using a 100-permutation test (35% SUVmax threshold P-MTV value with 40-cutpoint had a two-sided 100-permutation *p*-value = 0.02 – Monte Carlo 95% CI: 0.0001–0.0474). In women, depending on whether T-MTV was ≥ or <40 cm^3^, the 2-year EFS was 46.7% versus 86.9% respectively (Figure 1A), whereas in men it was 25.0% versus 50.0% (Figure 1B).

## 3. Discussion

To our knowledge, this retrospective study shows the prognostic value of total tumour metabolic volumes (primary lesion associated with lymph node involvement) in the initial assessment of locally advanced anal SCC using the largest number of patients to date. In addition, the originality of this study is based on the determination of these volumes according to different segmentation methods. This was done using either the fixed SUV thresholds commonly used in routine clinical practice, or mathematical methods of determining the optimal threshold by iterative approaches. Whilst the mathematical methods are generally less used, they may be available depending on the image-processing computer used. Our aim was to define the best and easiest method for application into clinical routine use with the possibility in the near future of defining personalized treatment (consolidation treatment or close observation) according to the prognosis index found.

After comparing primary tumour metabolic (P-MTV) and total tumour (T-MTV) volumes, the study identified T-MTV as the best independent prognostic factor for EFS. This corroborates with the results of Bazan et al., who showed using a smaller cohort, that T-MTV was also an independent prognostic factor, with an optimal threshold of 26 cm^3^ being much lower than that defined in our study at 40 cm^3^ [20]. This difference could be explained by the difference in sensitivity of the PET-CT used in the two studies (Bazan et al. used previous generation PET-CT cameras). On the other hand, Mohammadkhani Shali et al. recently reported that T-MTV was a negative prognostic factor at the threshold of 45 cm^3^ [31]. In this study, they examined a cohort of patients where some showed more advanced stages of disease (e.g., metastasis) compared to our study, and included metastatic involvement when this existed. Taken together, when all sites of initial disease are included, the T-MTV threshold lies between 40–45 cm^3^ to allow prognostic EFS discrimination. This threshold should be sought as the baseline in routine patient screening to allow a clinician to better understand the patient disease and a possible pejorative evolution. The systematic routine determination of T-MTV would be a first step towards personalized medicine, and would promote close patient monitoring when a high T-MTV was found. For this to become routine, the T-MTV calculation method must be easy, fast, reproducible and with a thresholding method available on the most common image processing computers.

The originality of our study is that we analyzed different segmentation methods for T-MTV. We did not find any added value for the iterative segmentation methods compared to the fixed SUV thresholds routinely used. This may be due to primary anal SCC often having a large volume and a high contrast due to high FDG avidity. The segmentation methods, whether by simple or iterative thresholding, operate in a very favorable situation for both. It is therefore not possible to highlight an impact of iterative thresholding methods in this context but iterative methods are known to be all the more relevant when the tumour volumes are small (less than 4–5 cm^3^) which is not the case for our cohort [39,40]. Moreover, a primary anal SCC tumour with a high FDG avidity and contrast compared to tissue background noise, favors the T-MTV determination by fixed thresholds which do not consider the surrounding peritumour tissue. The iterative thresholding methods would probably have been more effective in the context of small lesions or in a low-contrast environment such as in lung cancer [41]. Finally, iterative methods are described as more relevant in the event of movement during acquisition, as in the case of lung locations, which is not the case with pelvic involvement [39]. It should also be noted that the methods using fixed or iterative thresholding do not perform optimally in cases of highly heterogeneous lesions (intra-lesional necrosis for example) [42], which is, however, rarely the case in anal SCC.

The studies listed in Table 4 show a quasi-exclusive use of fixed thresholds of SUV but with an extended panel of these thresholds (from 25% to 50%). In our study, considering the segmentation methods according to a fixed threshold (35%, 41%, 50% of SUVmax), a threshold of 35% of SUVmax correlated slightly better with EFS, and importantly, is a threshold that is readily available on image processing computers. In order to simplify semiquantification, can we satisfied with only looking for the SUVmax for prognostic evaluation? In our study, the primary anal tumour SUVmax was not correlated with EFS, and is similar to the results published by Deantonio et al. [29]. On the contrary, Kidd et al. demonstrated a prognostic value of tumour SUVmax on the EFS but without any real multivariate analysis [30]. SUVmax is considered to be the easiest and most frequently used criterion in clinical practice to qualify a primary lesion, whereas this semiquantitative criterion, most often, represents only very limited information in considering radiotracer accumulation and not considering information on the associated tumour uptake distribution or on the overall tumour functional volume [43]. The two most recent studies that have assessed the prognostic value of FDG-PET scanning for primary anal SCC have used radiomics data and clinicopathological features [33,44]. Rusten et al. showed that PET metrics are not independent prognostic factors compared to clinicopathological factors. Brown et al. showed that the combination of radiomics and clinical data for their cohort of patients was the most efficient for predicting Progression Free Survival (PFS) on the initial and training cohorts with an AUC of 0.7412 and 0.7381, respectively. In the future, PET radiomics for anal SCC performed on larger cohorts will certainly improve the prognostic evaluation. 

It is interesting that the majority of published studies have focused on the metabolic volume of the primary tumour (P-MTV) and found this to be prognostic for EFS [30,33,34], and in one study for OS [28]. In our study, whilst both T-MTV and P-MTV were prognostic, T-MTV was more relevant.

**Figure 2:**
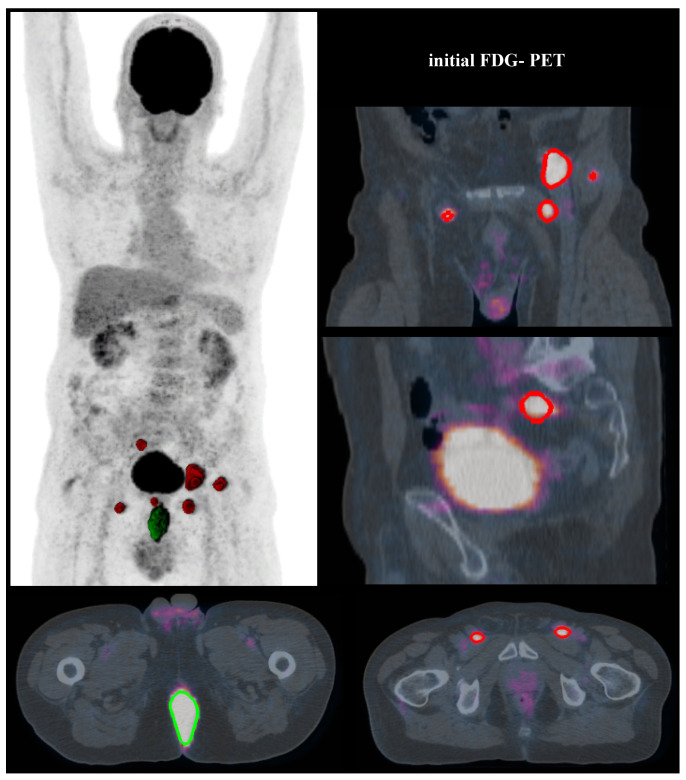
A T4N0 anal SCC was diagnosed at a 71-year-old patient. Initial FDG-PET modified the tumour staging as T4N3 due to an observed extended lymph node involvement (bilateral inguinal, perirectal and external iliac positive nodes). The T-MTV was 70.1 cm^3^ (with 35% SUVmax threshold). Imaging showed residual disease three months after the end of treatment, confirmed by pathological results of surgery. (Segmented volumes: for tumour in green, for lymph nodes in red).

An important finding of our study is that FDG-PET visual analysis of bilateral pelvic lymph node involvement resulted in a pejorative independent prognostic factor (*p* = 0.018), whereas in previous studies this was only the case for the presence or not of inguinal lymph node involvement [34]. The improved precision in our study is due to the classification of six distinct anatomical areas that include all of the potential lymph node lesions, allowing precise localized analysis. In addition, in our study, FDG-PET modified lymph node staging for 34.6% of the patients (Figure 2), which is similar to the results of a recent meta-analysis that changed lymph node status in 38% of patients [45]. Collectively, these data agree with the French national and European recommendations [4,5] which include carrying out an FDG-PET in the pretherapeutic assessment in order to diagnose pelvic or inguinal lymph node involvement not suspected by conventional imaging. They also align with the Current National Comprehensive Cancer Network guidelines to take into account the lymph node status evaluated by FDG-PET as part of the therapeutic strategy [46].

Whilst one limitation of our study lies in its retrospective nature, only three studies have been prospective [29,33,34], and the low incidence of this disease makes it difficult to carry out prospective studies. We analyzed a fairly large cohort of 81 patients treated according to standard protocols. However, these are preliminary data that require a larger number of patients to validate these data. Despite the FDG-PET acquisitions being carried out using two different PET-CT systems, no significant difference was found between the two PET-CT sites and the results allowed both qualitative and semiquantitative analysis.

## 4. Materials and Methods

### 4.1. Patients

Data from patients who underwent pretherapeutic FDG-PET as part of the extension assessment of their localized anal SCC were analyzed retrospectively (Table 1). The study was approved by the Institutional Ethics Committee (2019–27) and was conducted in accordance with the MR004 Reference Methodology. Patients were informed of the potential use of their data for research purposes. All patients had newly diagnosed and histologically proven anal SCC, and had not been treated. The exclusion criteria of the study were: another histological type of anal SCC, a cancer of the anal margin, a metastatic evolution (stage IV) or the existence of a second regional cancer such as prostatic or gynecological. The conventional pretreatment assessment included an endorectal echography and/or pelvic MRI and thoracoabdominopelvic CT for all patients, which allowed patient staging [40]. All patients received external pelvic curative radiotherapy ± extension to the inguinal lymph nodes (35.2 to 61.2 Gy), and was associated with chemotherapy in 68 patients (5-Fluorouracil-Mitomocycin C for 50 patients, 5-Fluorouracil-Cisplatin for 9 patients, and Capecitabine alone for 9 patients). Only one patient received neoadjuvant chemotherapy prior to concomitant chemoradiotherapy. Seventy-five patients received complementary irradiation (10 to 38 Gy) to the initial tumour volume (by external radiotherapy in 74 patients and by brachytherapy in 1 patient). At the end of the follow-up, an abdominoperineal resection was carried out on 21 patients: 19 of them due to an insufficient response to the therapeutic sequence, and for two others, due to radiotherapy-induced side effects such as pain and anal incontinence.

### 4.2. FDG-PET Acquisition

**Figure 3:**
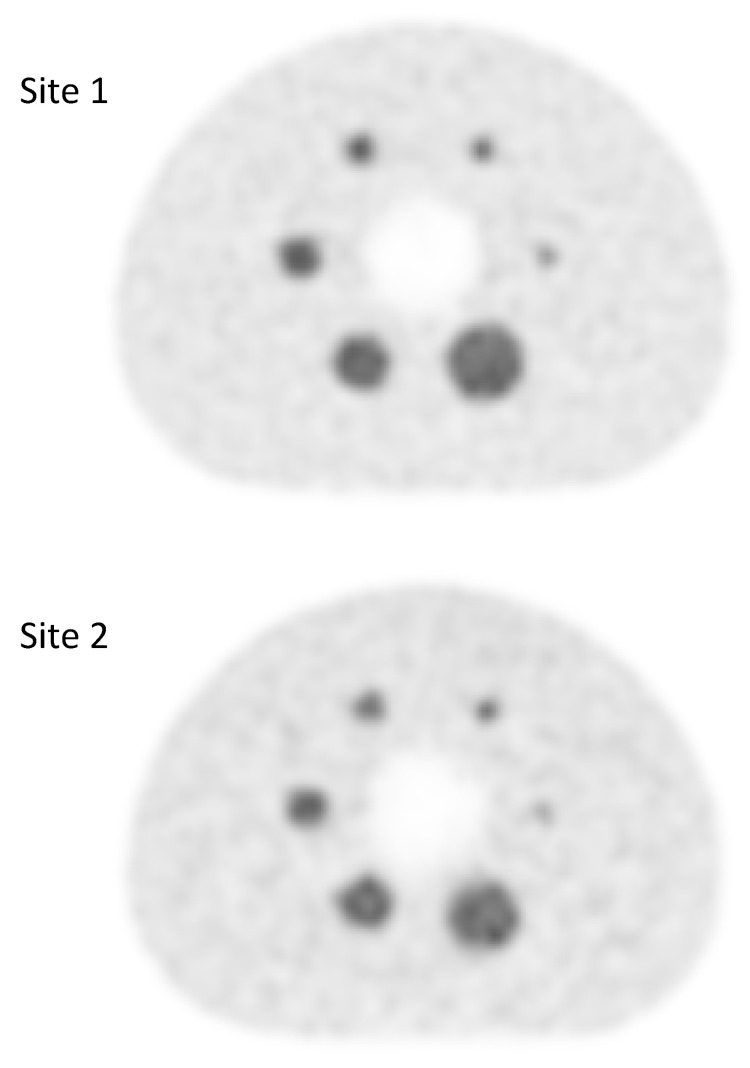
Qualitative assessment of FDG PET images obtained at site 1 (GE Discovery 690 PET scanner) and at site 2 (Siemens Biograph mCT 64 PET scanner).

**Figure 4:**
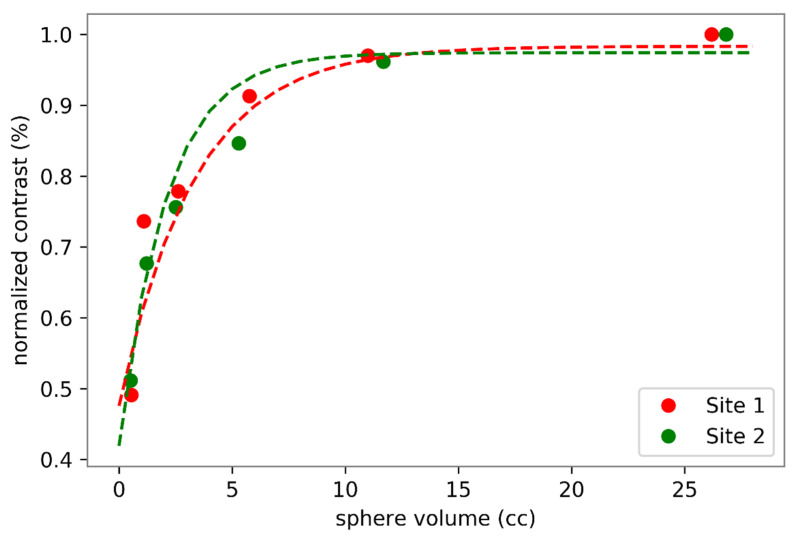
Quantitative assessment of FDG PET images obtained at site 1 (GE Discovery 690 PET scanner) and at site 2 (Siemens Biograph mCT 64 PET scanner). Sphere volumes correspond to sphere diameters of 10, 13, 17, 22, 28 and 37 mm.

The eighty-one examinations were performed using two types of PET-CT scanner. Forty-four with a Discovery 690 (GE Healthcare, Chicago, IL, USA) PET-CT system, and 37 with a Siemens Biograph mCT 64 (Siemens Healthcare Molecular Imaging USA Inc., Malvern, PA, USA). Because the PET-CT equipment in our institution is located at two different sites, it was necessary to evaluate any differences in contrast recovery coefficients of the reconstructed images to allow semiquantitative data pooling. This evaluation was carried out using a NEMA 2012-IEC 2008 phantom equipped with six spheres of 10, 13, 17, 22, 28, 37 mm in diameter. These six spheres were filled with an FDG activity concentration 5 times higher than the one filled in the tank. The images obtained at the two sites were reconstructed with the standard reconstruction parameters for each of the systems. A visual analysis did not show any difference (Figure 3), which was confirmed by the analysis of the recovery contrast curve (Figure 4). After a fast of at least 4 hours, 3 MBq/kg of FDG was injected intravenously. The acquisition was performed 60 ± 5 min after injection, from the base of the skull to the thighs, with the arms held above the head. The low-dose CT was performed according to a standardized protocol and preceded the PET acquisition (OSEM iterative reconstruction algorithm, two iterations, 24 subsets).

### 4.3. Interpretation of PET-CT Images and Segmentation Methods

The interpretation was carried out by two senior nuclear physicians without the results of conventional imaging. If the independent assessments were different, a consensus was reached after discussion.

For qualitative tumour and lymph node extension analysis, any focus of metabolic intensity greater than the hepatic background and correlated with a morphological lesion on CT was considered as positive. The loco-regional lymph node extension was classified according to six distinct anatomical areas: perirectal, left and right internal iliac, left and right inguinal and the other lymph node lesions more distant from the primary tumour (external and primitive iliacs) were grouped together.

A semiquantitative analysis of FDG-PET images was performed on a dedicated processing console (PlanetOnco, DOSIsoft, France). Segmentation of hypermetabolic foci was performed first by using standard segmentation methods followed by iterative approaches. Of the available fixed threshold methods for determining the lesion segmentation, we selected the three most commonly used thresholds reported in the literature (35%, 41% and 50% of the SUVmax). Four segmentation methods based on iterative approaches to determine the optimal threshold were used; adaptive [35], fitting [36], Black [37] and Nestlé methods [38]. The optimal segmentation threshold was defined using the intensity of the intralesional uptake and the surrounding background noise. These segmentation approaches first required a calibration procedure with a test object.

After identifying all hypermetabolic foci, we measured the Primitive Tumour Metabolic Volume (P-MTV) and the Total MTV (T-MTV) calculated as the sum of P-MTV and lymph node MTV. These metabolic volumes were measured with each of the segmentation methods. The SUVmax (maximum standardized uptake value) of the anal tumour was also collected.

### 4.4. Patient Follow-Up

Patients were considered as non-responders to the radio-chemotherapy sequence in case of residual disease (viable tumour within 6 months after treatment) or disease recurrence based on appearance of new lesions confirmed by follow-up and/or pathological data (from biopsies or surgical samples).

### 4.5. Statistical Analysis

Qualitative variables were described by the frequency of their respective modalities. Continuous variables were described by the median and its range (or mean ± SD). The evolutionary variables were described by the Kaplan–Meier method.

The primary endpoint was the event-free survival (EFS), defined as the time between the treatment start date and the date of the appearance of an event: residual disease, local recurrence or detection of distant metastasis. The gold standard for classification was based on clinical and/or imaging follow-up then by histological data when available (biopsy or surgical data). The median duration of patient follow-up was calculated using the inverse Kaplan–Meier method.

At univariate step, we determined if constitutive criteria (age, sex), conventional imaging (tumour and lymph node stage) and criteria derived from the qualitative analysis of FDG-PET (lymph node involvement, or bilateral node involvement) as well as semiquantitative data (SUVmax of the anal tumour and metabolic tumour volumes) had any prognostic value on EFS. For qualitative variables, log-rank test was used and for continuous variables, univariate Cox model. The aim was keeping only strong standard prognostic parameters for multivariate analysis. The set of variables associated with EFS at the 0.05 threshold in the univariate analysis were introduced into a multivariate Cox model. Several models were tested in order to introduce into each one only the clinical variables selected, and only one of the "independent" PET variables. The models were then compared by their AIC (Akaike Information Criterion). Optimal cutoff value determination was done using the Contal and O’Quigley method. Internal validation of final model prognostic parameter was done using a 100 Monte Carlo permutation test (Figure S1). All statistical analyses were two-sided, and *p* < 0.05 was regarded as statistically significant. The software used was Stata 13.1 Special Edition (StataCorp LP, College Station, TX, USA) and SAS 9.4 -TS1M2 (SAS Institute Inc., Cary, NC, USA).

## 5. Conclusions

Quantitation of the total tumour mass by using a fixed threshold on pretherapeutic FDG-PET in anal cancer is a valid tool for predicting EFS. If these data are confirmed in larger studies, FDG-PET could contribute to individualized patient therapies.

## Figures and Tables

**Table 1:** Patient characteristics.

Characteristics	*n* = 81
Negative HIV status	79 (97.5%)
HPV status	
Positive	22 (27.2%)
Negative	1 (0.2%)
Unknown	58 (71.6%)
Median age, years (range)	62.3 (32–89)
Sex	
Male	12 (14.8%)
Female	69 (85.2%)
TNM Classification with CI	
T1	6 (7.4%)
T2	32(39.5%)
T3	24 (29.6%)
T4	19 (23.5%)
N0	33 (40.7%)
N1	30 (37%)
N2	12 (14.8%)
N3	3 (7.4%)
Treatment	
Exclusive radiotherapy	13 (16%)
Radiotherapy + chemotherapy	68 (84%)
Surgery by APA	21 (25.9%)
Lymph node status established by pretherapeutic FDG PET	
N0	27 (33.3%)
N1	19 (23.5%)
N2	20 (24.7%)
N3	15 (18.5%)

HIV: Human Immunodeficiency Virus; CI: Conventional Imaging; APA: Abdomino-perineal amputation.

**Table 2:** Univariate Analysis.

Variables		HR	95% IC	*p* Values
Age		1.215	0.562–2.626	0.620
Sex (Male vs. Female)		3.326	1.379–8.020	**0.007**
T defined by conventional imaging (≥3 vs. 1 or 2)		1.648	0.747–3.638	0.216
N defined by conventional imaging (≥1 vs. 0)		1.657	0.720–3.813	0.235
N defined by FDG-PET (≥1 vs. 0)		2.316	0.873–6.145	0.092
Inguinal lymph node involvement defined on FDG-PET data		2.137	0.980–4.660	0.056
Bilateral pelvic lymph node involvement defined on FDG-PET data		2.764	1.194–6.398	**0.018**
Primitive tumour SUVmax		1.039	0.973–1.110	0.257
**50% SUVmax threshold**	**P-MTV**	1.030	1.009–1.052	**0.004 (0.061)**
	**T-MTV**	1.020	1.009–1.032	**<0.001 (0.006)**
41% SUVmax threshold	P-MTV	1.020	1.007–1.034	**0.003 (0.040)**
	T-MTV	1.020	1.009–1.032	**<0.001 (0.006)**
**35% SUVmax threshold**	**P-MTV**	1.018	1.007–1.029	**0.001 (0.018)**
	**T-MTV**	1.018	1.008–1.026	**<0.001 (0.003)**
Black threshold	P-MTV	1.012	1.005–1.019	**0.001 (0.017)**
	T-MTV	1.011	1.005–1.018	**<0.001 (0.004)**
**Adaptative threshold**	**P-MTV**	1.014	1.006–1.022	**0.001 (0.015)**
	**T-MTV**	1.014	1.006–1.021	**<0.001 (0.003)**
Nestlé threshold	P-MTV	1.014	1.005–1.022	**0.001 (0.016)**
	T-MTV	1.013	1.006–1.020	**<0.001 (0.004)**
**Fitting threshold**	**P-MTV**	1.016	1.007–1.016	**0.001 (0.015)**
	**T-MTV**	1.016	1.007–1.025	**<0.001 (0.004)**

HR: Cox Hazard Ratio; 95% CI: 95% Confidence Interval; T: Tumour; N: Node. For metabolic volume parameters, adjusted *p*-values were noted (bonferroni method); bold for the number of digits given.

**Table 3:** Multivariate Cox analysis for pretreatment MTV (P-MTV and T-MTV) associations with EFS. Bold indicates a statistically significant association.

Models	Variables	HR	*p*	95% IC	AIC	Models	Variables	HR	*p*	95% IC	AIC
**1**	Male sex	3.351	**0.007**	1.383–8.122		**1′**	Male sex	3.513	**0.006**	1.437–8.591	
**P-MTV_50_**		1.030	**0.012** **(0.16)**	1.006–1.053	205.5708	**T-MTV_50_**		1.021	**0.002 (0.028)**	1.008–1.034	202.7038
	BPNI	2.192	0.170	0.934–5.143			BPNI	1.948	0.129	0.824–4.607	
**2**	Male sex	3.371	**0.007**	1.389–8.184		**2′**	Male sex	3.513	**0.006**	1.437–8.591	
**P-MTV_41_**		1.021	**0.006 (0.084)**	1.006–1.036	204.6024	**T-MTV_41_**		1.021	**0.002 (0.028)**	1.437–8.591	202.7038
	BPNI	2.278	0.057	0.976–5.317			BPNI	1.948	0.129	0.824–4.607	
**3**	Male sex	3.647	**0.005**	1.489–8.934		**3′**	Male sex	3.799	**0.004**	1.538–9.384	
**P-MTV_35_**		1.018	**0.003 (0.042)**	1.006–1.031	203.9462	**T-MTV_35_**		1.018	**0.001 (0.014)**	1.007–1.028	202.0206
	BPNI	2.026	0.106	0.861–4.769			BPNI	1.736	0.215	0.726–4.150	
**4**	Male sex	3.946	**0.003**	1.587–9.814	203.7218	**4′**	Male sex	4.031	**0.003**	1.613–10.072	
**P-MTV_black_**		1.012	**0.003 (0.042)**	1.004–1.021		**T-MTV_black_**		1.012	**0.001 (0.014)**	1.005–1.019	202.0069
	BPNI	1.859	0.170	0.766–4.510			BPNI	1.711	0.237	0.702–4.167	
**5**	Male sex	3.838	**0.004**	1.551–9.498	203.3572	**5′**	Male sex	3.883	**0.004**	1.562–9.653	
**P-MTV_adaptative_**		1.015	**0.002 (0.028)**	1.005–1.024		**T-MTV_adaptative_**		1.014	**0.001 (0.014)**	1.006–1.022	201.5438
	BPNI	1.987	0.121	0.834–4.735			BPNI	1.750	0.216	0.721–4.249	
**6**	Male sex	3.877	**0.003**	1.564–9.609		**6′**	Male sex	3.900	**0.003**	1.567–9.700	
**P-MTV_Nestlé_**		1.014	**0.002 (0.028)**	1.005–1.023	203.3357	**T-MTV_Nestlé_**		1.014	**0.001 (0.014)**	1.006–1.022	201.9119
	BPNI	1.952	0.133	0.834–4.735			BPNI	1.759	0.213	0.723–4.278	
**7**	Male sex	3.755	**0.004**	1.525–9.246		**7′**	Male sex	3.818	**0.004**	1.543–9.444	
**P-MTV_Fitting_**		1.017	**0.003 (0.042)**	1.006–1.028	203.7705	**T-MTV_Fitting_**		1.016	**0.001 (0.014)**	1.006–1.026	202.4940
	BPNI	1.878	0.162	0.776–4.548			BPNI	1.728	0.231	0.706–4.232	

HR: Cox Hazard Ratio; 95% CI: 95% Confidence Interval; AIC: Akaike Information Criterion; P-MTV: Metabolic Tumour Volume of primitive tumour; T-MTV: Metabolic Tumour Volume of primitive tumour and pelvic involved nodes; BPNI: Bilateral pelvic node involvement. For metabolic volume parameters, adjusted *p*-values were added (Bonferroni method).

**Table 4:** Literature review. Prognostic performances of pretherapeutic FDG-PET in patients with anal SCC cancer.

Authors	Year	Study Status	N	Thresholds of SUV_max_	Pronostic Value of Pretreatment FDG PET
Kidd et al. [24]	2010	R	77	-	SUVmax > 5.6 predicts: - increased lymph node metastases (*p* < 0.0001) - worse 2-year PFS (*p* = 0.05) - increased risk of persistent or recurrent disease on post-therapy FDG-PET performed <4 mo post-treatment completion (*p* = 0.0402)
Bazan et al. [18]	2013	R	39	≥50% of SUV_max_	Total-MTV (>26 cm^3^) is an independent prognostic factor for PFS (*p* = 0.01) and EFS (*p* = 0.02).
Deantonio et al. [23]	2015	*p*	55	≥2.5 of SUV	SUVmax was not prognostic for survival outcomes.
Gauthé et al. [22]	2016	R	75	≥50% of SUV_max_	Patients with Primitive-MTV_50_ > 7 cm^3^ had worse OS (*p* = 0.028).
Mohammadkhani Shali et al. [25]	2016	R	45	≥30% SUV_max_	Patients with MTV_30_ (primitive tumour + node + metastases)> 45 cm^3^ had higher risk of recurrence (*p* = 0.019).
Duimering at al. [34]	2019	P	73	≥25%, 40% and 50% of SUV_max_	Patients with MTV_25_ > 35 cm^3^ had worse PFS (*p* = 0.011) and CSS (*p* = 0.024).
Rusten et al. [33]	2019	P	93	≥2.5 of SUV	Combination of HPV and ZMP, MTV, or TLG performed equally well as combination of HPV and N3 to predict PFS (*p* < 0.02).
Brown et al. [44]	2019	R	189	t-VOI or LN-VOI > 1.5 times Liver-VOI	Combined radiomic/clinical prognostic factors provide better PFS (AUC 0.7412) than conventional staging parameters.
**Our Study**	**2019**	**R**	**81**	**≥35%, 41% and 50% of SUV_max_ and 4 iterative methods (Black, adaptative, Nestlé and Fitting)**	**Whatever the SUV_max_ threshold, patients with Total-MTV (primitive tumour + pelvic nodes) > 40 cm^3^ had worse EFS (*p* = 0.0015).**

NA: non-applicable; R: retrospective study; P: prospective study; PFS: progression free survival; OS: overall survival; EFS: event-free survival; CSS: cause-specific survival; ZMP: Z-normalized combination of MTV and SUVpeak.

## Supplementary Materials

The following are available online at https://www.mdpi.com/2072-6694/12/6/1512/s1, Figure S1: Monte Carlo permutation test.
