# Supplementary material for: Prognostic Impact of Pretherapeutic FDG-PET in Localized Anal Cancer
Source: Cancers (Basel). 2020 Jun 9;12(6):1512. doi: 10.3390/cancers12061512 (PMC7352672; doi:10.3390/cancers12061512)
Supplement: Supplementary file 1 [file cancers-12-01512-s001.pdf]

cancers

MDPI

Article

Prognostic Impact of Pretherapeutic FDG-PET in Localized Anal Cancer

Maelle Le Thiec, Aude Testard, Ludovic Ferrer, Camille Guillerminet, Olivier Morel, Bruno Maucherat, Daniela Rusu, Sylvie Girault, Marie Lacombe, Hadji Hamidou, Véronique Guérin Meyer, Emmanuel Rio, Sandrine Hiret, Françoise Kraeber-Bodéré, Loïc Campion and Caroline Rousseau

Final Result

Cut-Points		Contal and O'Quigley Method					Cox Model Hazard Ratio		Cox Model Wald P-value		False Discovery Rate	
Cut Level	VOL_TOT_T1_MAX35	SK	Absolute SK	Q Statistic	P-value	Selected Cut-Point	Hazard Ratio	Selected Cut-Point	P-value	Selected Cut-Point	P-value	Selected Cut-Point
1	5	5.0580266	5.0580266	1.0989469	0.1787		.		.		.	
2	10	5.4203951	5.4203951	1.1776779	0.1248		3.683		0.0338		0.0465982	
3	15	4.8456299	4.8456299	1.0527999	0.2179		2.276		0.0632		0.0680334	
4	20	4.4941704	4.4941704	0.976439	0.3000		2.018		0.0777		0.0776816	
5	25	5.2491157	5.2491157	1.1404644	0.1484		2.283		0.0381		0.0465982	
6	30	6.2902553	6.2902553	1.3666706	0.0477		2.902		0.0069		0.0120345	
7	35	6.3995302	6.3995302	1.3904126	0.0419		3.170		0.0036		0.010145	
8	40	6.4962794	6.4962794	1.411433	0.0372	<====	3.578		0.0015		0.0052271	<====
9	45	6.37816	6.37816	1.3857695	0.0430		3.963		0.0007		0.0052271	<====
10	50	5.9604841	5.9604841	1.2950219	0.0699		4.142	<====	0.0007	<====	0.0052271	<====
11	55	5.3534495	5.3534495	1.1631328	0.1336		4.071		0.0012		0.0052271	<====
12	60	4.0003886	4.0003886	0.8691561	0.3000		3.681		0.0057		0.0114546	
13	65	4.0003886	4.0003886	0.8691561	0.3000		3.681		0.0057		0.0114546	
14	70	3.076858	3.076858	0.6685025	0.3000		3.023		0.0277		0.0430402	
15	75	2.5356767	2.5356767	0.5509212	0.3000		3.082		0.0399		0.0465982	

Figure S1. Monte Carlo permutation test.
